# Identification of Genomic Loci Associated with *Rhodococcus equi* Susceptibility in Foals

**DOI:** 10.1371/journal.pone.0098710

**Published:** 2014-06-03

**Authors:** Cole M. McQueen, Ryan Doan, Scott V. Dindot, Jessica R. Bourquin, Zlatomir Z. Zlatev, M. Keith Chaffin, Glenn P. Blodgett, Ivan Ivanov, Noah D. Cohen

**Affiliations:** 1 Department of Large Animal Clinical Sciences, Texas A&M University College of Veterinary Medicine & Biomedical Sciences, College Station, Texas, United States of America; 2 Department of Veterinary Pathobiology, Texas A&M University College of Veterinary Medicine & Biomedical Sciences, College Station, Texas, United States of America; 3 Department of Molecular and Cellular Medicine, Texas A&M University College of Medicine, College Station, Texas, United States of America; 4 Department of Electrical and Computer Engineering, Texas A&M University Dwight Look College of Engineering, College Station, Texas, United States of America; 5 6666 Ranch, 1102 Dash For Cash Road, Guthrie, Texas, United States of America; 6 Department of Veterinary Physiology & Pharmacology, Texas A&M University College of Veterinary Medicine & Biomedical Sciences, College Station, Texas, United States of America; The University of Melbourne, Australia

## Abstract

Pneumonia caused by *Rhodococcus equi* is a common cause of disease and death in foals. Although agent and environmental factors contribute to the incidence of this disease, the genetic factors influencing the clinical outcomes of *R. equi* pneumonia are ill-defined. Here, we performed independent single nucleotide polymorphism (SNP)- and copy number variant (CNV)-based genome-wide association studies to identify genomic loci associated with *R. equi* pneumonia in foals. Foals at a large Quarter Horse breeding farm were categorized into 3 groups: 1) foals with *R. equi* pneumonia (clinical group [N = 43]); 2) foals with ultrasonographic evidence of pulmonary lesions that never developed clinical signs of pneumonia (subclinical group [N = 156]); and, 3) foals without clinical signs or ultrasonographic evidence of pneumonia (unaffected group [N = 49]). From each group, 24 foals were randomly selected and used for independent SNP- and CNV-based genome-wide association studies (GWAS). The SNP-based GWAS identified a region on chromosome 26 that had moderate evidence of association with *R. equi* pneumonia when comparing clinical and subclinical foals. A joint analysis including all study foals revealed a 3- to 4-fold increase in odds of disease for a homozygous SNP within the associated region when comparing the clinical group with either of the other 2 groups of foals or their combination. The region contains the transient receptor potential cation channel, subfamily M, member 2 (*TRPM2*) gene, which is involved in neutrophil function. No associations were identified in the CNV-based GWAS. Collectively, these data identify a region on chromosome 26 associated with *R. equi* pneumonia in foals, providing evidence that genetic factors may indeed contribute to this important disease of foals.

## Introduction

*Rhodococcus equi* is an important intracellular pathogen affecting horses, most commonly among foals in which it causes chronic, suppurative bronchopneumonia [1], as well as extrapulmonary disorders [2]. The cumulative incidence of pneumonia caused by *R. equi* may be high at breeding farms with affected foals, and this disease may adversely impact future racing performance [3]. At affected farms, a varying proportion of foals will develop clinical signs of pneumonia while the other foals remain free of the disease; however, subclinical pneumonia can occur following either experimental or natural infection with *R. equi* [4]–[6].

Although the factors contributing to *R. equi* pneumonia are complex, recent evidence suggests that some horses may be genetically predisposed to this condition [1], [7], [8]. Identifying the genetic and biological basis of susceptibility, or perhaps resistance, to *R. equi* pneumonia in foals is important, because it might lead to the development of diagnostic and therapeutic tools to manage at-risk foals on breeding farms and might shed light on critical host defense mechanisms. Currently, single nucleotide polymorphism (SNP)-based genotyping platforms are available for performing genome-wide association studies (GWAS) in horses [9]. Use of SNP-based genotyping platforms to identify genomic regions associated with particular phenotypes in animals is growing at a rapid pace [9]–[14]. As a result, researchers, veterinarians, and producers increasingly rely on data from these studies to make important production and management decisions [15], [16].

Although high-density SNP arrays are powerful tools for performing association studies, they are often inadequate for examining structurally complex regions, particularly those enriched with copy number variants (CNVs) [17]. Results from the 1000 Genomes project estimate approximately 20% of CNVs are not in linkage disequilibrium with flanking or tagging SNPs [18], indicating that additional testing is required to accurately genotype these variants. The identification of CNVs is further complicated by the probe placement and design of most commercial SNP arrays [17]–[19]. Comparative genomic hybridization (CGH) arrays are optimized for genotyping CNVs. Using SNP and CGH arrays together may, in some instances, increase the power of a GWAS by expanding the number of informative markers, particularly within structurally complex regions of the genome [19].

In horses, CNVs are present in genes involved in many biological processes and may underlie or modify many common and disease traits [20]–[22]. Of the CNVs in horses identified to date, most are enriched in genes involved in sensory perception, signal transduction, and metabolism [22]. In other animal species, including horses, CNVs often affect genes regulating the immune system, particularly the MHC; they may also be causative or modifying variants of many immune related conditions [23]–[30].

The genetic basis of susceptibility or resistance to *R. equi* pneumonia has not been explored on a genome-wide basis. Here, we describe independent SNP- and CNV-based GWAS to identify genomic loci associated with *R. equi* pneumonia in Quarter Horse foals. We identified a number of regions associated with *R. equi* pneumonia, including a region on chromosome 26. Located within this region is the transient receptor potential cation channel, subfamily M, member 2 (*TRPM2*) gene that encodes a protein associated with neutrophil function.

## Materials and Methods

### Ethics statement

All protocols for this study were reviewed and approved by the Clinical Research Review Committee (CRRC Protocol 10–12), College of Veterinary Medicine & Biomedical Sciences, Texas A&M University. This study was carried out on private land (33°37′14″N 100°19′22″W) and specific permissions for use were granted by GPB. During the time this study was conducted, research involving client-owned animals at Texas A&M University was not subject to review by the Institutional Animal Care and Use Committee. Written informed consent for participation was obtained for all foals included in the study, and the 6666 Ranch provided access to the foals included in this project. This study did not involve any endangered or protected species.

### Study population

The 6666 Ranch was selected as the site for this study because it agreed to provide access to foals, had history of *R. equi* pneumonia among foals with a cumulative incidence of ≥15% for the preceding 3 years, and because the farm's veterinarian/general manager (GPB) was conducting a separate study during 2011 evaluating screening tests for *R. equi* pneumonia in foals, which was directed by one of the authors (MKC). The screening test evaluation required that treatment was not initiated for any foal on the basis of screening test results alone, and that the veterinarians making decisions about diagnosis and treatment of *R. equi* pneumonia were not informed of the results of screening tests. Each foal at the farm underwent bilateral thoracic ultrasonographic examination at 2-week intervals, beginning at 3 weeks of age either until 19 weeks of age or until the foal developed clinical signs of pneumonia (as described below). Ultrasonographic examinations were performed by a veterinarian who did not participate in diagnosis or treatment of *R. equi* pneumonia. The anatomic location (left versus right hemithorax; intercostal space; and, dorsal, middle, or ventral region) and maximal diameter of any areas of pulmonary abscesses or consolidation were recorded. In addition, the total number of lesions was counted.

All foals [N = 248] born at the farm during 2011 were eligible to be included in the study. All foals were monitored daily by farm personnel for clinical signs of pneumonia until 20 weeks of age. Clinical signs suggestive of pneumonia included fever, lethargy, signs of depressed attitude, cough, nasal discharge, polysynovitis, tachypnea, increased respiratory effort, respiratory distress, and detection of a tracheal rattle or pulmonary crackles or wheezes via thoracic auscultation. For each foal that developed clinical signs of pneumonia, thoracic ultrasonography and collection of a trans-endoscopic tracheobronchial aspirate (TBA) sample with a commercially available triple-guarded catheter (Triple stage tracheal wash catheter, MILA International Inc., Erlanger, KY) were performed. Between uses, the endoscope was disinfected with a 3.4% glutaraldehyde solution (CIDEX-PLUS, Advanced Sterilization Products, Irvine, CA) following a standard protocol used in our laboratory and known to be microbicidal against *R. equi*. Each sample of TBA fluid was submitted for microbiologic culture and cytologic evaluation to the Texas Veterinary Medical Diagnostic Laboratory in College Station, Texas.

Foals with *R. equi* pneumonia (clinical group; N = 43 [17%]) were defined as those having signs of pneumonia at 3 to 20 weeks of age, ultrasonographic evidence of peripheral pulmonary consolidation or abscesses at the time of examination for clinical signs of pneumonia, and *R. equi* detected in TBA fluid via microbiologic culture, and cytological evidence of gram-positive intracellular coccobacilli in the TBA sample. Subclinical foals (N = 156 [63%]) were defined as those having ultrasonographic evidence of peripheral pulmonary consolidation or abscesses, but lacking clinical signs of pneumonia [31]. Unaffected foals (N = 49 [20%]) were classified as having no clinical signs of pneumonia and no ultrasonographic evidence of pulmonary consolidation or abscessation. From each of the 3 groups of foals (i.e., clinical, subclinical, and unaffected), 24 foals were selected randomly for the SNP- and CNV-based genome-wide association studies. The rationale for including 24 foals was based on funding available to conduct the study (rather than an *a priori* sample size calculation).

### DNA samples and isolation

A blood sample (4 mL) was collected by jugular venipuncture into a tube containing acid citrate dextrose (ACD) as an anticoagulant from the first (i.e., age 3 weeks) blood sample obtained from each foal. Genomic DNA was isolated using a standard phenol-chloroform isoamyl extraction protocol from these blood samples from each foal [22].

### SNP genotyping and data analysis

The SNP genotyping was performed at Gene Seek (Neogen, Lincoln, NE) using the EquineSNP70 BeadChip Array (Illumina, San Diego, CA). The resulting SNP genotypes were analyzed using the PLINK analysis package [32]. Genotypes were determined for each animal and then filtered (i.e., excluded) on the basis of missingness per individual (>10%), missingness per SNP (>10%) minor allele frequency (<5%), and absence of Hardy-Weinberg equilibrium (P<0.001), as described by Raudsepp *et al*. [12]. A standard chi-square association test (Max(T) permutations [N = 10,000]) based on a binary outcome of disease status using a case-control design was performed using PLINK [12], [33]. Genotype ped files were loaded into PLINK and foals were assigned a phenotypic status of either affected or unaffected (case/control). A P value of P<1×10^−5^ was considered evidence of association [34]. Population stratification was determined using plots of the observed versus the expected -log_10_ P values of Cochran-Armitage trend tests and by determining the genomic inflation factor, λ, using the R package *GenABEL* [35]. Using the R package pedigreemm [36], mixed-effects logistic regression with sire modeled as a random effect was used for the association test in comparisons showing evidence of population stratification; SNPs with any genotype represented fewer than 10 times were removed from analysis to permit model convergence [35]–[37]. All SNP array data have been deposited in NCBI's Gene Expression Omnibus (GEO) [38] and are accessible through GEO Series accession number GSE57510 (http://www.ncbi.nlm.nih.gov/geo/query/acc.cgi?acc=GSE57510).

### Joint analysis

Genotyping for the joint analysis was performed using a tetra-primer AMRS PCR genotyping reaction [39] of an individual SNP (SNP ID:UKUL3936) present on the EquineSNP70 array and located within the *TRPM2* gene (Forward outer: 5′-ATCAGCCAGACACTCCAGGCATGACAT-3′; Forward inner: 5′-CATCCTCCTCAGCCACCTGCATCTTTT-3′; Reverse outer: 5′-ATCTCAGAAGGAGCTGCCATGCCTACC-3′; and, Reverse inner: 5′-GTATCTTCAGGACCACCCTCCTGACGC-3′). The primers were designed using Primer3 software [40] and synthesized by Sigma-Aldrich (St. Louis, MO). The PCR reactions were performed under these conditions: 9.8 µl mili-Q H_2_0, 4 µl Taq FlexiBuffer (Promega, Madison, WI), 2 µl MgCl_2_, 0.4 µl dNTPs, 0.1 µl Taq (Promega), 1 µl forward and reverse inner primer, 0.1 µl forward and reverse outer primer, and 1.5 µl of DNA at 50 µg/µl. Cycling conditions were as follows: 94°C for 2 m; 35 cycles at 94°C for 1 m; 65.8°C for 1 m; 72°C for 1 m; and, a final extension at 72°C for 2 m. PCR amplicons were resolved on a 2% agarose gel.

Genotype data from the joint analysis were analyzed using logistic regression on the basis of the binary outcome of disease (pneumonia versus each of the respective comparison groups [i.e., clinical foals, subclinical foals, and unaffected foals]). The association of disease with genotype for the SNP was expressed as the odds ratio (OR), estimated from logistic regression modeling; 95% confidence intervals were estimated using maximum likelihood methods. Models were fit for comparisons of clinical versus subclinical foals, clinical versus the combination of subclinical and unaffected foals, and clinical versus unaffected foals. Models were fit using S-PLUS statistical software (Version 8.2, TIBCO, Inc., Seattle, WA). A significance level of P<0.05 was used for the analyses.

### CNV detection and analyses

Copy number variants were identified using a previously reported equine exome array for comparative genomic hybridization (CGH)[41]. An individual Quarter Horse mare was used as the reference sample for each CGH experiment [22]. Array CGH was performed using methods described by Doan *et al*. [41]. Briefly, genomic DNA was sonicated and then labeled with the Cy5 (experimental) and Cy3 (reference) AlexaFluor dyes using the BioPrime Plus labeling kit (Invitrogen, Carlsbad, CA). Two µg of reference and experimental DNA were hybridized onto the arrays (Agilent Technologies, Santa Clara, CA). The arrays were scanned using an Agilent DNA Microarray Scanner (2-µm settings and 0.05 XDR). Fluorescent intensity values were calculated using Agilent's Feature Extraction 10.5 software (Agilent). Copy number variants, including their corresponding log_2_ ratios, were identified using Agilent's Genomics Workbench 7.

Copy number variants were called using the ADM-2 algorithm and the following filters: minimum probe span ≥3 and average log_2_ ratio ≥0.5, removal of probes with ≥3 standard deviations above or below the mean log_2_ fluorescent intensity.

Logistic regression modeling was used to perform 2 separate CNV-based GWAS. The first approach modeled the association of the binary outcome of 2 groups (e.g., clinical versus subclinical) with the log_2_ ratio of intensity values (a continuous variable) for each CNV. The second approach modeled the association of the binary outcome of 2 groups with the presence or absence of a CNV (a binary categorical variable) within a CNV region. For the first approach, CNV regions (CNVRs) were determined for the foals examined. The CNVRs were then filtered to include only CNVs identified in at least 3 foals. The log_2_ ratios of probes within each CNVR were then averaged to calculate a single log_2_ (CNVR-log_2_ ratio) for each CNVR for each foal. The CNVR-log_2_ values were used in a logistic regression model to identify associated CNVs among the pairwise comparisons of the 3 groups (case-control design described above). For the second approach, a logistic regression model involving the binary outcome of presence (or absence) of a CNV within each CNVR was used to identify associated CNVs among the pairwise comparisons of the 3 groups. For both approaches, the generated P values from linear modeling or regression analyses were corrected for multiple comparisons using the method outlined by Hochberg *et al*. [42]. Statistical analyses were performed using R (Version 3.0.1; R Statistical Project). All CNV data have been deposited in NCBI's Gene Expression Omnibus (GEO) [38] and are accessible through GEO Series accession number GSE57510 (http://www.ncbi.nlm.nih.gov/geo/query/acc.cgi?acc=GSE57510).

## Results

### SNP-based GWAS

**Figure 1:**
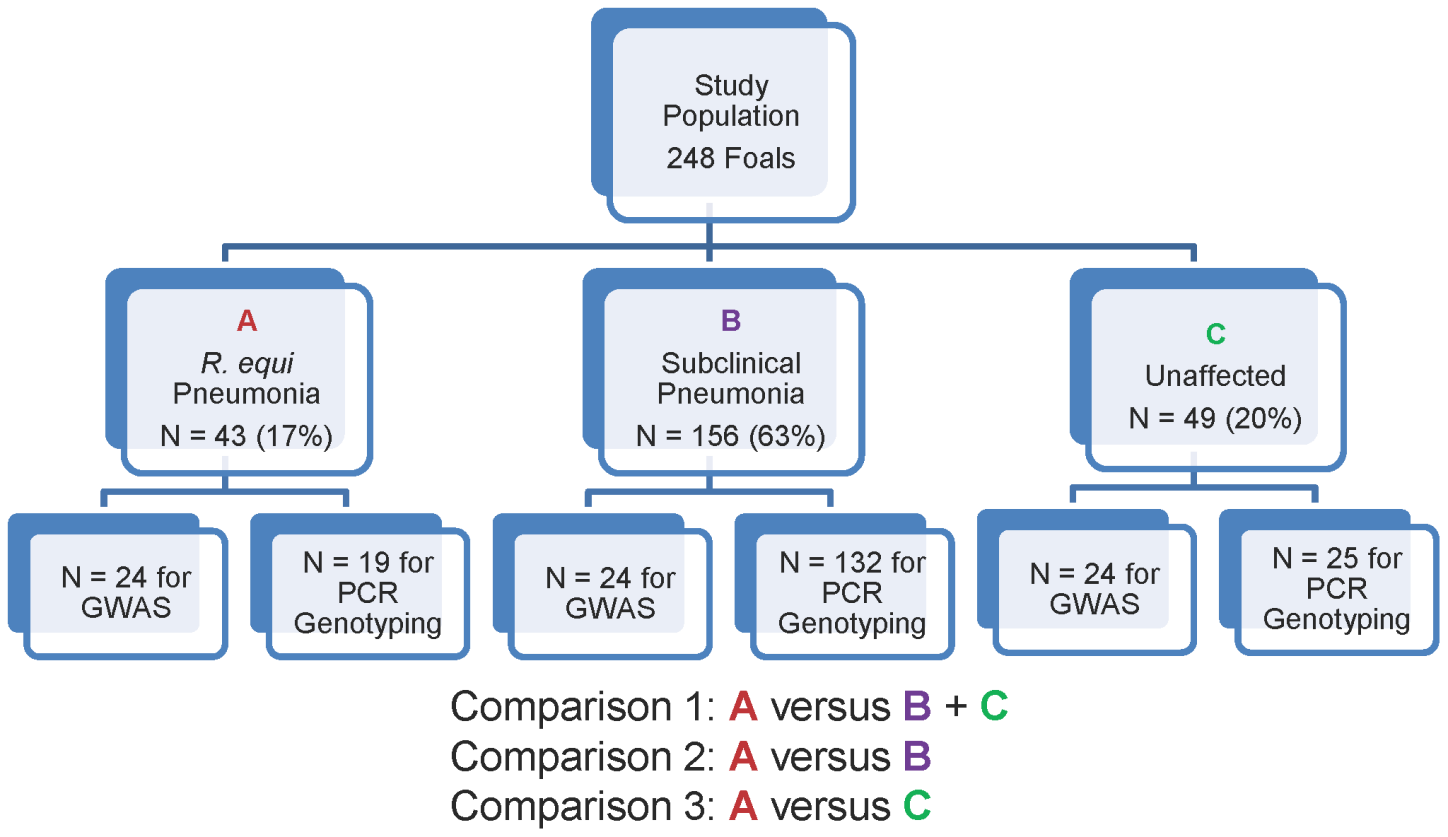
Schematic diagram representing the distribution of the total population into the 3 subgroups (*R. equi* pneumonia foals [clinical], subclinical foals, and unaffected foals), and by genome-wide association studies versus PCR genotyping for TRPM2 SNP. The 3 comparisons among groups are also summarized.

**Figure 2:**
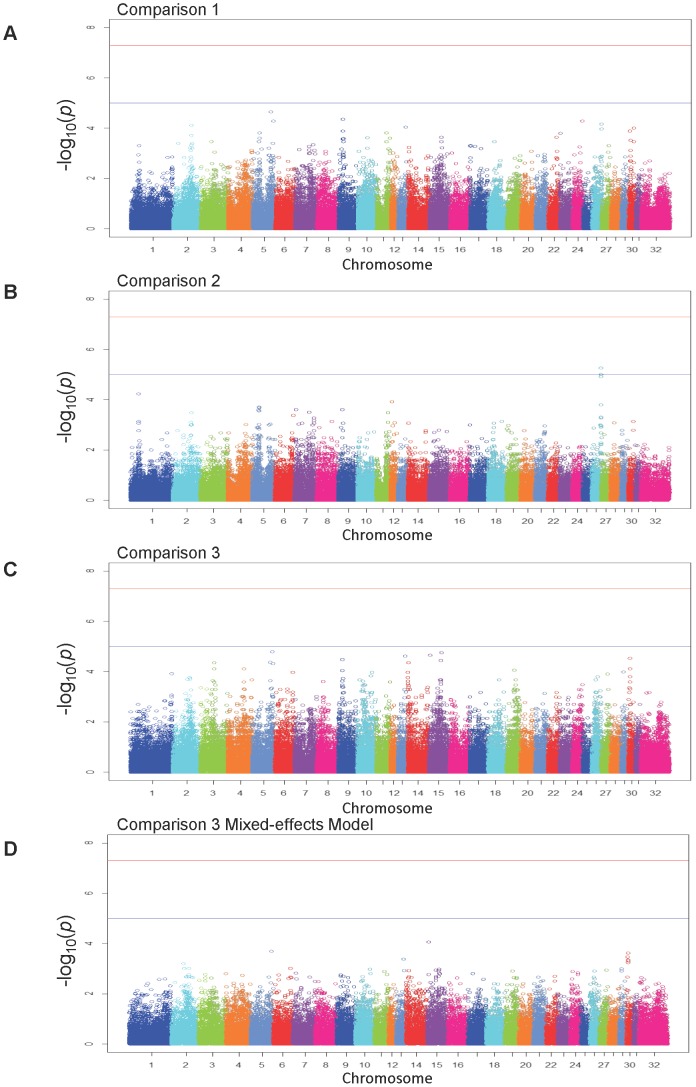
Manhattan plots of standard chi-squared significance values for the 3 genome-wide association studies. Manhattan plots for (A) comparison 1, (B) comparison 2, and (C) comparison 3; (D) Mixed effects-model analysis of comparison 3.

Three case-control GWAS were performed among the 24 randomly selected foals representing each group (clinical group, subclinical group, and unaffected group; **Figure 1**). The number of SNPs excluded on the basis of missingness per individual, missingness per SNP, and minor allele frequency were 0, 1,292, and 11,157, respectively (12,449 total SNPs). For comparisons 1, 2, and 3 (Figure 1), the number of SNPs excluded on the basis of Hardy-Weinberg equilibrium were 0, 83, and 54, respectively. After filtering, the total genotyping rate of the foals was estimated at 99.4%. Comparison 1 (clinical [N = 24] vs. subclinical + unaffected [N = 48]; λ = 1.16) identified 7 regions showing evidence of moderate association with clinical pneumonia (P<1×10^−5^) (**Figure 2A** **and** **Table 1**). Comparison 2 (clinical [N = 24] vs. subclinical [N = 24]; λ = 1.00 [**Figure S1A**]) identified 10 regions with moderate association (**Figure 2B** **and** **Table 1**). The region associated with clinical pneumonia had a (point-wise) value of EMP1 ≤0.0002. Comparison 3 (clinical [N = 24] vs. unaffected [N = 24]; λ = 1.44 [**Figure S1B**]) identified 2 regions with moderate association (**Figure 2C** **and** **Table 1**). Results from each GWAS comparison are provided in Table S1.

**Table 1:** Moderately associated SNPs for each genome-wide association study.

**Comparison 1**			
**SNP ID**	**Chromosomal location**	**P Value**	**Odds Ratio**
BIEC2_921509	5:79290169	2.21E-05	0.2
BIEC2_1137861	9:19578673	4.42E-05	9.556
BIEC2_976727	5:91479639	5.10E-05	0.09677
BIEC2_650473	24:41033593	5.16E-05	0.04274
BIEC2_732054	26:39777632	6.78E-05	12.05
BIEC2_696979	26:39861109	6.78E-05	12.05
BIEC2_696992	26:39867963	6.78E-05	12.05
BIEC2_492054	2:80197359	7.73E-05	0.2036
BIEC2_240596	13:31572932	8.94E-05	0.156
BIEC2_825623	30:20687203	9.79E-05	0.2369
BIEC2_867975	30:20690855	9.79E-05	0.2369
**Comparison 2**			
**SNP ID**	**Chromosomal location**	**P Value**	**Odds Ratio**
BIEC2_732054	26:39777632	5.54E-06	17.89
UKUL3936	26:39640172	9.93E-06	12.69
BIEC2_696979	26:39861109	1.24E-05	16.43
BIEC2_696992	26:39867963	1.24E-05	16.43
BIEC2_16162	1:35593058	5.80E-05	13.8
**Comparison 3**			
**SNP ID**	**Chromosomal location**	**P Value**	**Odds Ratio**
BIEC2_927543	5:88617368	1.63E-05	0.1486
BIEC2_311792	15:54419913	1.75E-05	0.1515
BIEC2_284540	15:7394044	2.21E-05	0.08571
BIEC2_240596	13:31572932	2.41E-05	0.1163
BIEC2_858227	30:6939831	2.99E-05	0.1512
BIEC2_1078504	9:19582539	3.37E-05	0.1314
BIEC2_310113	15:51485703	3.57E-05	0.1074
BIEC2_310214	15:51596762	3.57E-05	0.1074
BIEC2_324522	15:51634407	3.57E-05	0.1074
BIEC2_921509	5:79290169	4.19E-05	0.1667
BIEC2_784299	3:59782881	4.45E-05	0.1445
BIEC2_239002	14:3055273	4.46E-05	0.1696
BIEC2_976312	5:90703095	4.80E-05	0.09333
BIEC2_928796	5:90706671	4.80E-05	0.09333
BIEC2-815626	30:5321760	7.70E-05	0.1169
BIEC2_784331	3:59874438	7.87E-05	5.8
BIEC2_784332	3:59874495	7.87E-05	5.8
BIEC2_869756	4:72076243	7.87E-05	5.8
BIEC2_435811	19:33380654	8.85E-05	0.1784
BIEC2_1137861	9:19578673	9.20E-05	10.09

The λ value of comparison 3 (1.44) suggested evidence of confounding population structure from 1 of the groups, thus mixed-effects modeling with sire as the random effect term was used as an additional association test. There were 23,318 SNPs filtered due to failure to converge in mixed modeling, leaving 40,843 SNPs for evaluation in comparison 3. The logistic mixed-effects modeling reduced the λ from 1.44 to 1.10 (**Figure S1D**), and inspection of the observed versus expected P value plot indicated that none of the smallest P values [highest –log_10_ P values] observed were greater than expected (**Figure 2D** **and Table S2**). The mixed-effects model analysis identified 1 region with modest evidence of association (BIEC2_284540; chr15:7,394,044; P = 9.0×10^5^). The λ values of each comparison suggested the population structure was attributable to foals in the unaffected group because the magnitude of λ was greatest for the comparison of clinical versus unaffected groups (i.e., strongest evidence of population stratification), was 1.00 for clinical versus subclinical groups (i.e., absence of population stratification), and 1.14 when the clinical group was compared with the combination of healthy and subclinical (indicating the healthy group contributed to evidence of population stratification for this comparison).

A region on chromosome 26 (chr26:39,640,172–39,867,963) showed evidence of association with clinical pneumonia (Comparisons 1 and 2), with the strongest evidence of association in comparison 2. Three of the 4 SNPs identified in this region were BIEC2_732054, BIEC2_696979, and BIEC2_696992, with the first lying in a keratin-associated protein (*KRTAP*) gene and the remaining 2 SNPs lying in non-genic locations. The region also contained a potential candidate gene (*TRPM2*) based on biological function [43] identified by the SNP marker, UKUL3936 (P = 9.93×10^−6^; OR = 12.7), which was also located within exon 22 of the *TRPM2* gene. On the basis of this finding, a joint analysis was performed to include results of PCR-based genotyping of the UKUL3936 SNP for the samples from the larger remaining population of foals not included in the GWAS study (i.e., clinical [N = 19], subclinical, [N = 132], and unaffected [N = 25]). Prior to performing the joint analysis, 10 of 72 foals previously genotyped on the SNP array were used to validate the genotyping reaction used for the joint analysis; results of SNP array and PCR genotyping agreed for all 10 foals. The joint analysis comparing clinical versus subclinical foals (comparison 2) revealed that foals from the clinical group were approximately 4-fold less likely to have either an AB (1/(0.23)  = 4.3; P = 0.0017) or BB genotype (1/(0.28)  = 3.6, P = 0.0574), consistent with a dominant model, possibly with partial penetrance (**Table 2**). Considering just the AA genotype relative to the other genotypes, the odds of disease were approximately 3.7-fold greater for foals with the AA genotype (Table 2; P = 0.0006). Using an additive model, there was a significant (P = 0.0014) association of the A allele in comparisons between the clinical and other groups, with an estimated odds of a clinical classification being increased nearly 3-fold for each copy of the A allele. Examination of the genotype data, however, suggested an additive model was unlikely: the ORs for the AB and BB types relative to the AA genotype were similar. The AA genotype of the TRPM2 gene also was significantly associated with increased odds of *R. equi* pneumonia when considering the results of comparisons of the clinical foals versus all foals (comparison 1; Table S2) and for clinical foals versus healthy foals (comparison 3; Table S3), irrespective of the genetic model.

**Table 2:** Joint analysis of *TRPM2* SNP UKUL3936.

Genotype				
*Standard Model*	Clinical foals	Subclinical foals	P value	Odds Ratio (95% CI)
AA	72% (31/43)	41% (64/156)	NA	1 (NA)
AB	23% (10/43)	47% (74/156)	0.0017	0.28 (0.13 to 0.61)
BB	5% (2/43)	12% (18/156)	0.0574	0.23 (0.05 to 1.04)
***Dominant Model***				
Not AA	28% (12/43)	59% (92/156)	NA	1 (NA)
AA	72% (31/43)	41% (64/156)	0.0006	3.71 (1.78 to 7.76)
***Additive Model***				
f(A)*	2(0 to 2)	1(0 to 2)	0.0014	2.83 (1.51 to 5.31)
	(72/86) 84%	(202/312) 65%		

*Median (range) reported for frequency of allele A, along with the proportion of A alleles among all alleles represented for each group. Joint analysis includes genotypes derived from SNP array and PCR genotyping.

### CNV-based GWAS

Next, array aCGH was performed to genotype CNVs in the 72 foals examined in the SNP-based GWAS. Two reactions failed to meet the minimum quality scores for CNV detection, so they were excluded from the study (foals 153 and 278). Collectively, 6,727 CNVs were identified among the 70 foals (**Table 3** **and Table S3**). Merging shared CNVs yielded 2,350 CNV regions (CNVR) that were present at 3,492 Ensembl annotated genes (3,442 protein-coding and 50 RNA-coding). The lengths of CNVs ranged from 197 base-pairs (bp) to 7,229.5 kilo-bp (kb), with a mean length of 97.7 kb, median length of 4.4 kb, and mode length of 960 bp.

**Table 3:** CNVs identified in the each clinical group.

	Clinical foals	Subclinical foals	Unaffected foals	P Value
**Total CNVs**	84 (29 to 592; 2,654)	57 (29 to 338; 1,823)	77 (28 to 254; 2,250)	0.262
**Gains**	25 (8 to 395; 1,229)	24 (9 to 187; 770)	30 (10 to 152; 957)	0.334
**Losses**	41 (15 to 197; 1,425)	36 (16 to 151; 1,053)	40 (16 to 139; 1,293)	0.574

CNVs: median (range; sum); Kruskal-Wallis test.

The association between disease status (the outcome variable) and individual CNVs (dependent variable) was assessed using logistic regression analysis. Because CNV genotypes reflect differences in DNA content between 2 individuals and are expressed as normalized log_2_ ratios representative of varying degrees of copy number gains and losses, separate logistic regression modeling was performed using CNVs as the dependent variable as either 1) continuous variables representing average log_2_ ratios of CNVRs or 2) a binary variable representing the presence or absence of a given CNV (see Materials and Methods section). Comparisons among groups of foals were made in the CNV-based GWAS as described above (**Figure 1**). Correction for multiple comparisons revealed no significant (P<0.05) associations of disease with CNVs when considered as continuous log_2_ ratios (**Table 4**) or as the binary outcome for presence or absence of a CNV. No association with clinical status grouping was detected on the basis of the total number of CNVs for individuals (**Table 3**).

**Table 4:** Top 5 CNV regions identified using logistic regression for the association of *R. equi* with either the binary variable presence or absence of a CNV identified in the region (Presence columns) or the log_2_ ratio of intensity values of the CNVs (Intensity columns).

Presence		Intensity (log_2_)	
CNVR	Location	CNVR	Location
232	chr1:158563545-160296515	179	chr1:135750728-135756968
237	chr1:161277315-161277884	391	chr2:88754970-89061174
269	chr2:6304157-6378969	458	chr3:36530104-36530453
536	chr4:21281685-23733388	645	chr5:40060212-40060524
735	chr6:42263179-42282649	753	chr6:59580727-59585139

CNVR identification numbers are provided in Supplementary Table 1.

## Discussion

*Rhoddococcus equi* is an important cause of disease and death in young foals [44]. Multiple factors such as age, environmental conditions including level of exposure to virulent organisms, and genetic background appear to play a role in the occurrence of this complex disease [45]–[47]. The purpose of this study was to better characterize the genetic basis of susceptibility to *R. equi* pneumonia. One special feature of this study was the phenotypic characterization of foals into those that remained unaffected (i.e., free of both clinical signs and ultrasonographic evidence of disease) through weaning, those that had subclinical pneumonia (i.e., absence of clinical signs but ultrasonographic evidence of pulmonary lesions), and those that developed *R. equi* pneumonia. At most farms where screening is performed, foals with evidence of subclinical pneumonia receive treatment or other interventions that precludes one from differentiating foals that would have progressed to clinical disease from those that would have remained subclinical. At the farm described in this report, however, foals with subclinical pneumonia were not treated or otherwise managed differently than unaffected foals providing us with the exceptional opportunity to conduct GWAS's with 3 clinically important phenotypes.

A SNP-based GWAS revealed a region on chromosome 26 (chr26:39640172–39867963) that was positively associated with disease. This region contains the *TRPM2* gene, which is associated with neutrophil function. Although, *TRPM2* is an ideal candidate based on its known biological function, an adjacent SNP located within the *KRTAP* gene was more strongly associated than the SNP located with *TRPM2*. Further, investigation of this region (e.g., fine-mapping of the region) is needed before any conclusion can be made regarding the role of *TRPM2* in *R. equi* pneumonia. Nevertheless, *TRPM2* is of interest because it has been demonstrated in mice to play a role in neutrophil-mediated tissue damage [43]. Neutrophils have been shown to play an important role in the outcome of *R. equi* infection. Neutrophil-depleted mice had significantly heavier tissue burdens of *R. equi* following experimental infection than non-depleted mice, documenting a protective role for neutrophils [48]. The neutrophil concentrations at 2 and 4 weeks of age were significantly lower among foals that subsequently developed *R. equi* pneumonia than among age-matched foals that did not develop pneumonia [49]. Similar protective roles for neutrophils have been documented for other intracellular pulmonary pathogens [48], [50], [51]. Moreover, *R. equi* has an age-dependent distribution (i.e., foals are usually affected and adults are generally resistant to infection), and age-related differences in neutrophil responses to *R. equi* have been documented [52]–[54]. Although neutrophils play a role in protecting against *R. equi* infection, they also contribute to lung parenchymal damage of this pyogranulomatous disease [44], [55]. In a mouse model of ulcerative colitis, the over-abundance of neutrophil invasion into tissue mediated by *TRPM2* expression, led to increased colonocyte death [43]. Thus, variation in *TRPM2* expression could influence the extent to which neutrophil-induced pulmonary damage occurs following infection with *R. equi*, and this variation could be a crucial determinant of the clinical outcome of infection with *R. equi* and the progression from subclinical to clinical pneumonia. If the *TRPM2* allele implicated in our study were associated with increased *TRPM2* expression, it might consequently be associated with greater likelihood of pneumonia development as a result of greater neutrophil invasion. The functional effects of this *TRPM2* genotype are unknown, however. Interestingly, expression of a splice variant of *TRPM2* was demonstrated to inhibit death of several cultured cell lines [56]. Further evaluation of the functional differences in neutrophilic responses among *TRPM2* genotypes is warranted.

A CNV-based GWAS was conducted and revealed no significant association with disease status. Despite the negative findings, these results are of interest with regard to better characterizing genetic variation in horses and using CNVs for GWAS with disease outcomes. A search of PubMed reveals no other attempt to perform a CNV GWAS via aCGH in horses and to the authors' knowledge no others have been done. Valuable information was gained in terms of the analysis of CNV data generated from a GWAS using aCGH. The identification of CNVs is based on log_2_ ratios of intensity signals that are generated between the reference and sample DNA. The results may be interpreted either as presence or absence of a CNV, based on a threshold intensity value [41], or the actual intensity values themselves. As observed in this study, these 2 outcomes for CNV-calling yielded differing results. We propose that utilizing average log_2_ ratios across CNV regions is superior because classification of CNVRs by presence or absence does not further characterize a CNV, whereas analysis of the log_2_ ratios allows for the identification of whether the CNV involved gains or losses, and for description of the magnitude of the gain or loss.

We failed to identify significant association with candidate genes previously associated with *R. equi* pneumonia in other breeds of foals [1], [7], [8]. This may have been attributable to differences among populations of foals studied (e.g, breeds) or study methodology (e.g, case definitions, methods for detecting polymorphisms, etc.). Nevertheless, a commonality among these studies can be found in their identification of genes pertaining to host defenses against infectious pathogens, such as iron transport and innate immune responses. Conceivably, these apparently discrepant findings may converge on critical biological pathways or processes that influence susceptibility to infection with *R. equi* (and other intracellular pathogens).

This study had a number of limitations. First, it was likely underpowered for both the SNP- and CNV-based GWAS portions of the study: we only had the opportunity to find SNPs or CNVs with large effects. This lack of power is probably why we did not identify highly significant associations in the SNP-based GWAS for 2 of our 3 comparisons (Figure 1), and only moderate significance for the allele identified in comparison 2. Although sample sizes may be calculated for human studies, methods for incorporating crucial determinants of sample size such as the impact of the relatively longer length of linkage disequilibrium in horses relative to humans remain to be defined. Moreover, the cost of GWAS studies and the limited funding available for equine research can be restrictive. It is worth noting that our SNP-based GWAS did provide sufficient power to identify a SNP associated with *R. equi* that was subsequently substantiated by findings of the joint analysis using PCR testing of additional foals from this population for all 3 comparisons. Nevertheless, larger scale studies are indicated. For example, 1 locus on chromosome 15 (in the promoter region of a chemokine) was weakly associated in both the mixed-effects modeling and standard GWAS analyses for comparison 3: this finding could be simply attributable to chance but also could represent an underpowered association that merits further investigation.

A limitation of this study is that it was restricted to foals of a single breed at a single farm. Further studies are indicated to substantiate whether the observed association holds among other Quarter Horse foals at other farms, and among foals of other breeds. Another limitation was that although the mixed modeling for comparison 3 yielded a marked reduction in the estimated value of λ (from 1.44 to 1.10), the mixed modeling value of λ suggested residual confounding from population structure. Graphical analysis of the results of mixed modeling, however, suggested this was not the case: the plot of observed versus expected P values indicated that fewer than expected small P values were identified and more than expected large P values were observed following mixed-effects modeling, and Manhattan plots of the standard and mixed-effects model GWAS for comparison 3 revealed a shift to larger P values following mixed-effects modeling (**Figure S1**). Moreover, the linear regression-based estimate of λ had a poor fit because the data were non-linear in the range of the high P values (data not shown). We interpreted these results to indicate that the mixed-effects modeling had largely corrected for population stratification attributable to sire, and that the study was underpowered. As noted, a limitation of this study is that we lack fine-mapping of the region to determine whether the *TRPM2* allele is a causal variant or simply in linkage disequilibrium with another gene or genes that contribute to susceptibility to *R. equi*. Finally, it must be noted that although the estimated OR for the *TRMP2* SNP was relatively large, it is not of sufficient magnitude to be clinically useful for screening purposes.

Despite the aforementioned limitations, this study has identified a region on chromosome 26 that was associated with *R. equi* pneumonia in foals, and the largest scale GWAS in foals reported to date. Furthermore, it extends current knowledge of equine CNVs and analysis of data from GWAS using aCGH in horses.

## Conclusions

In conclusion, the results of this study identify a locus and gene potentially involved in the development of *R. equi* pneumonia in foals. Future studies are warranted to substantiate the association of *TRPM2* gene (and related pathways) with *R. equi* pneumonia and to provide fine-mapping of the region on chromosome 26 implicated in this GWAS.

## Acknowledgments

We acknowledge the 6666 Ranch for providing access to the foals studied; Ms. Ellen Ruth Alexander for help with the PCR genotyping; and, Mr. Nick Culliton for assisting with the DNA extractions.

## Supporting Information

Figure S1 **Quantile-quantile (QQ) plots of expected chi-squared significance values plotted against the observed values.** QQ plots for (A) comparison 1, (B) comparison 2, (C) comparison 3, and (D) mixed-effects model.
(TIFF)

Table S1 **CNV regions called from foals across all phenotypic groups during CNV association analysis.**
(XLSX)

Table S2 **Results of joint analysis comparing clinical foals with the combined subclinical and unaffected foals (comparison 1).**
(DOCX)

Table S3 **Results of joint analysis comparing clinical foals with unaffected foals (comparison 3).**
(DOCX)
