# Supplementary figures and images for: Identification of Genomic Loci Associated with Rhodococcus equi Susceptibility in Foals
Source: PLoS One. 2014 Jun 3;9(6):e98710. doi: 10.1371/journal.pone.0098710 (PMC4043894; doi:10.1371/journal.pone.0098710)

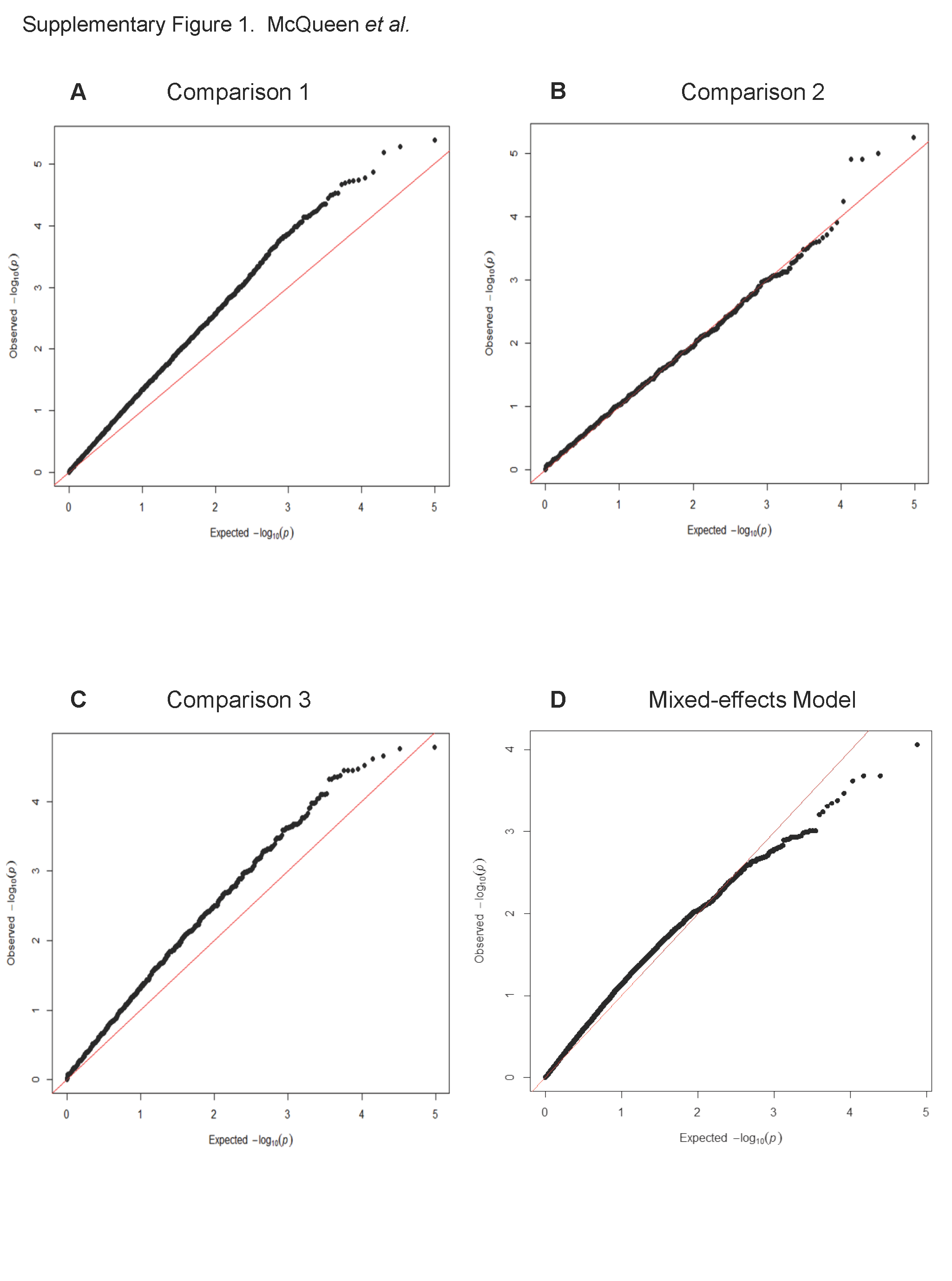

Supplement: Figure S1 — Quantile-quantile (QQ) plots of expected chi-squared significance values plotted against the observed values. QQ plots for (A) comparison 1, (B) comparison 2, (C) comparison 3, and (D) mixed-effects model. (TIFF) [file pone.0098710.s001.tiff]
