# Supplementary material for: Identification of Genomic Loci Associated with Rhodococcus equi Susceptibility in Foals
Source: PLoS One. 2014 Jun 3;9(6):e98710. doi: 10.1371/journal.pone.0098710 (PMC4043894; doi:10.1371/journal.pone.0098710)
Supplement: Table S2 — Results of joint analysis comparing clinical foals with the combined subclinical and unaffected foals (comparison 1). (DOCX) [file pone.0098710.s003.docx]

Supplementary Tables

| **Supplementary Table 2.** Joint analysis of *TRPM2* SNP UKUL3936 (Comparison 1) | | | | |
| --- | --- | --- | --- | --- |
| **Genotype** |  | | | |
| ***Standard Model*** | **Clinical foals** | **Subclinical + Unaffected foals** | **P value** | **Odds Ratio (95% CI)** |
| AA | 72% (31/43) | 43% (89/205) | NA | 1 (NA) |
| AB | 23% (10/43) | 46% (95/205) | 0.0016 | 0.29 (0.11 to 0.62) |
| BB | 5% (2/43) | 11% (21/205) | 0.0826 | 0.27 (0.06 to 1.18) |
|  | | | | |
| ***Dominant Model*** |  | | | |
| Not AA | 28% (12/43) | 57% (116/205) | NA | 1 (NA) |
| AA | 72% (31/43) | 43% ( 89/205) | 0.0007 | 3.50 (1.71 to 7.16) |
|  | | | | |
| ***Additive Model*** |  | | | |
| f(A)* | 2(0 to 2) | 1(0 to 2) | 0.0019 | 2.71 (1.45 to 5.06) |
|  | 84% (72/86) | 66% (271/410) |  |  |
| * Median (range) reported for frequency of allele A, along with the proportion of A alleles among all alleles represented for each group. Joint analysis includes genotypes derived from SNP array and PCR genotyping. | | | | |
