# Supplementary material for: Identification of Genomic Loci Associated with Rhodococcus equi Susceptibility in Foals
Source: PLoS One. 2014 Jun 3;9(6):e98710. doi: 10.1371/journal.pone.0098710 (PMC4043894; doi:10.1371/journal.pone.0098710)
Supplement: Table S3 — Results of joint analysis comparing clinical foals with unaffected foals (comparison 3). (DOCX) [file pone.0098710.s004.docx]

| **Supplementary Table 3.** Joint analysis of *TRPM2* SNP UKUL3936 (Comparison 3) | | | | |
| --- | --- | --- | --- | --- |
| **Genotype** |  | | | |
| ***Standard Model*** | **Clinical foals** | **Unaffected foals** | **P value** | **Odds Ratio (95% CI)** |
| AA | 72% (31/43) | 47% (23/49) | NA | 1 (NA) |
| AB | 23% (10/43) | 47% (23/49) | 0.0177 | 0.32 (0.13 to 0.81) |
| BB | 5% (2/43) | 6% ( 3/49) | 0.4622 | 0.49 (0.08 to 3.20) |
|  | | | | |
| ***Dominant Model*** |  | | | |
| Not AA | 28% (12/43) | 47% (23/49) | NA | 1 (NA) |
| AA | 72% (31/43) | 53% (26/49) | 0.0179 | 2.92 (1.22 to 6.98) |
|  | | | | |
| ***Additive Model*** |  | | | |
| f(A)* | 2(0 to 2) | 1(0 to 2) | 0.0398 | 2.20 (1.05 to 4.64) |
|  | 84% (72/86) | 70% (69/98) |  |  |
| * Median (range) reported for frequency of allele A, along with the proportion of A alleles among all alleles represented for each group. Joint analysis includes genotypes derived from SNP array and PCR genotyping. | | | | |
